# Supplementary figures and images for: Spatio-temporal variation of Cerambycidae-host tree interaction networks
Source: PLoS One. 2020 Feb 10;15(2):e0228880. doi: 10.1371/journal.pone.0228880 (PMC7010308; doi:10.1371/journal.pone.0228880)

# Supporting information

S1 Fig. Beetle principal component analysis.

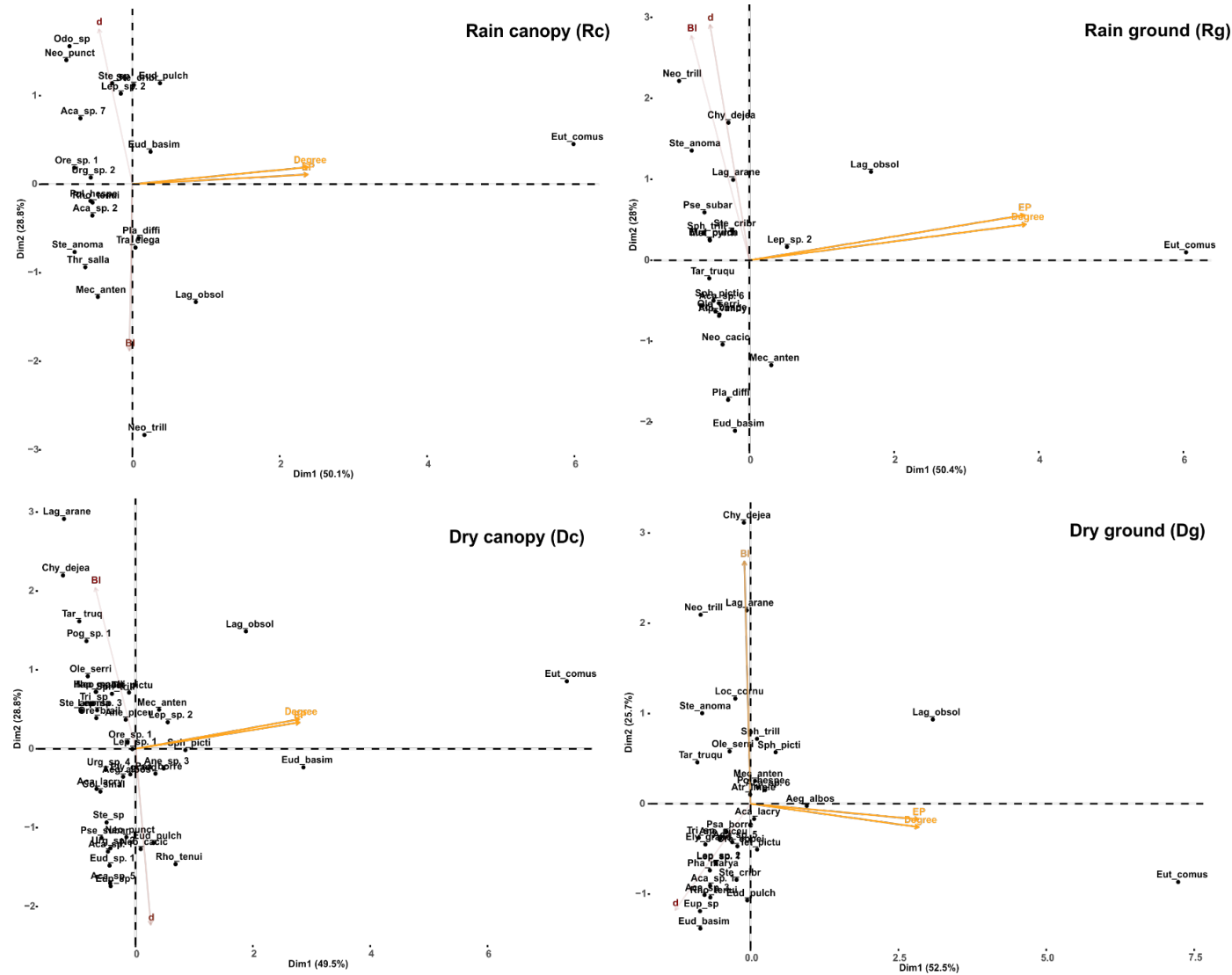

Supplement: S1 Fig — (PDF) [file pone.0228880.s001.pdf]

**S2 Fig. Host tree principal component analysis.**

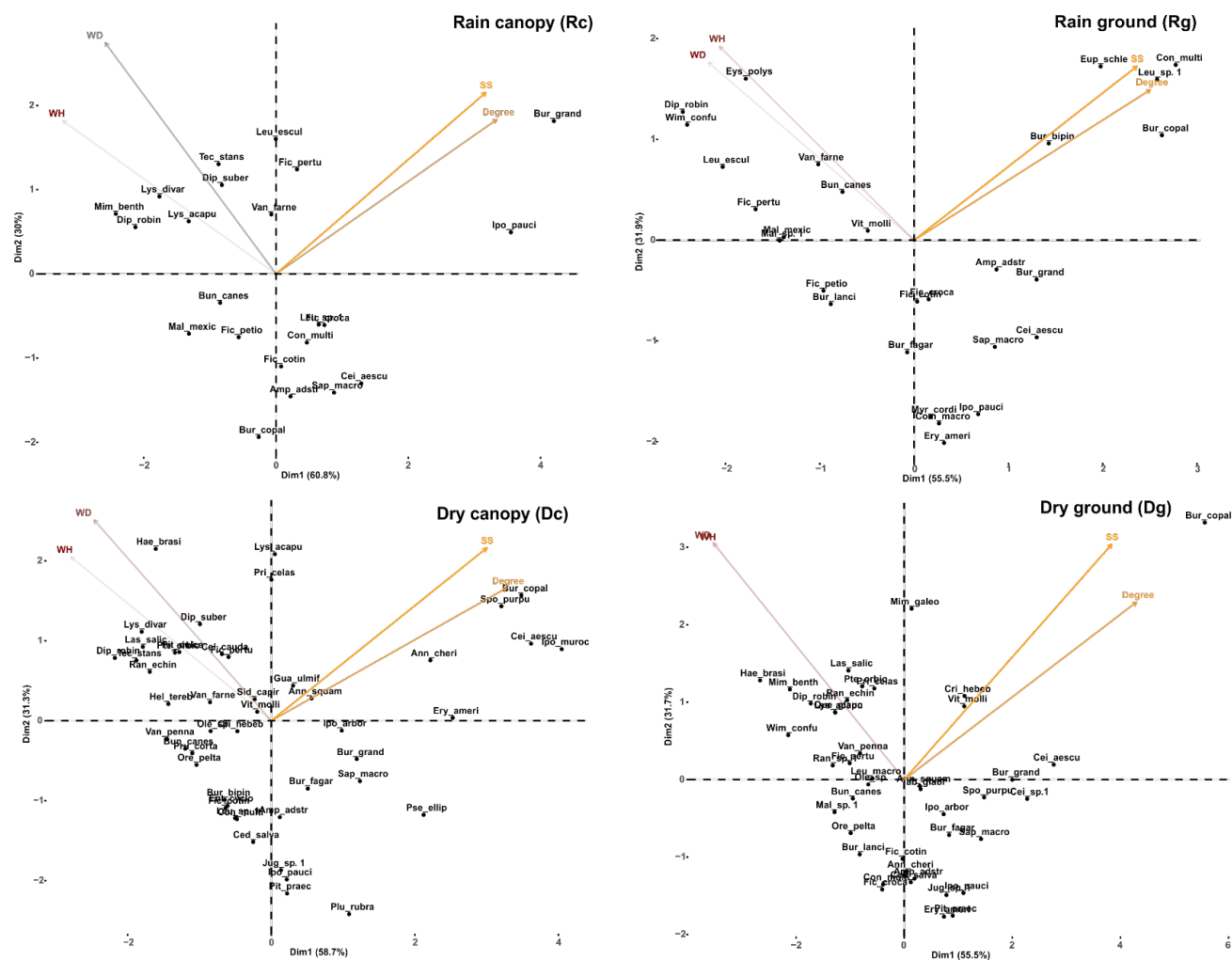

Supplement: S2 Fig — (PDF) [file pone.0228880.s002.pdf]
