# Supplementary material for: Spatio-temporal variation of Cerambycidae-host tree interaction networks
Source: PLoS One. 2020 Feb 10;15(2):e0228880. doi: 10.1371/journal.pone.0228880 (PMC7010308; doi:10.1371/journal.pone.0228880)
Supplement: S1 Table — Degree (number of interactions), d (species specialization), Species strength (SS), Effective partners (EP) and a measure of body size index (BSI) presented the average. (PDF) [file pone.0228880.s003.pdf]

Supporting information

S1 Table. Beetles species of tree-beetle network parameters per treatment in a tropical dry forest. Degree (number of interactions), d (species specialization), Species strength (SS), Effective partners (EP) and a measure of body size index (BSI) presented the average.

Rain canopy						
ID	Beetle species	Degree	d	SS	EP	BSI
Aca_sp. 2	Acantocinini sp. 2	1	0.46	0.13	1.00	27.05
Aca_sp. 7	Acantocinini sp. 7	1	0.72	0.33	1.00	10.54
Eud_basim	*Euderces basimaculatus* (Giesbert & Chemsak, 1997)	2	0.59	0.69	1.89	11.54
Eud_pulch	*Euderces pulchra* (Bates, 1874)	3	0.90	1.67	1.98	31.61
Eut_comus	*Eutrichillus comus* (Bates, 1881)	13	0.54	8.39	5.89	27.42
Lag_obsol	*Lagocheirus obsoletus obsoletus* (Thomson, 1860)	3	0.49	0.67	2.34	105.13
Lep_sp. 2	*Leptostylus* sp. 2	2	0.87	1.63	1.57	30.01
Mec_anten	*Mecotetartus antennatus* (Bates, 1872)	1	0.34	0.08	1.00	59.82
Neo_punct	*Neocompsa puncticollis asperula* (Bates, 1885)	1	1.00	1.00	1.00	29.65
Neo_trili	*Neoptychodes trilineatus* (Linnaeus, 1771)	2	0.41	0.39	1.65	181.32
Odo_sp	*Odontocera* sp.	1	0.94	0.83	1.00	6.83
Ore_sp. 1	*Oreodera* sp. 1	1	0.82	0.50	1.00	70.15
Pla_diffi	*Placosternus difficilis* (Chevrolat, 1862)	2	0.50	0.68	1.57	54.40
Pol_hespe	*Poliaenus hesperus* (Chemsak y Linsley, 1988)	1	0.50	0.15	1.00	25.04
Rho_tenui	*Rhopalophora tenuis* (Chevrolat, 1855)	1	0.46	0.13	1.00	17.42
Ste_anoma	*Steirastoma anomala* (Bates, 1880)	1	0.79	0.54	1.00	126.29
Ste_sp	*Stenolis* sp.	2	0.87	0.98	1.38	20.70
Ste_cribr	*Stenosphenus cribripennis cribripennis* (Thomson, 1860)	2	0.93	1.67	1.89	35.94
Thr_salla	*Thryallis sallaei* (Bates, 1880)	1	0.58	0.20	1.00	91.90
Tra_elega	*Trachyderes elegans blandus* (Dupont, 1840)	2	0.85	1.20	1.89	140.29
Urg_sp. 2	*Urgleptes* sp. 2	1	0.50	0.15	1.00	7.74
Rain ground						
ID	Beetle species	Degree	d	SS	EP	BSI
Aca_sp. 6	Acantocinini sp. 6	1	0.7	0.4	1	15.75
Alp_vandy	*Alphomorphus vandykei* (Linsley, 1930)	1	0.6	0.2	1	34.1
Chy_dejea	*Chyptodes dejeani* (Thomson, 1865)	2	0.7	1.2	1.9	183.9
Eud_basim	*Euderces basimaculatus* (Giesbert & Chemsak, 1997)	1	0.1	0	1	11.54
Eud_pulch	*Euderces pulchra* (Bates, 1874)	1	1	1	1	31.61
Eut_comus	*Eutrichillus comus* (Bates, 1881)	17	0.5	12	8.1	27.42
Lag_arane	*Lagocheirus araneiformis ypsilon* (Voet, 1778)	2	0.6	0.4	1.8	155.8
Lag_obsol	*Lagocheirus obsoletus obsoletus* (Thomson, 1860)	6	0.8	3.2	4	105.1
Lep_sp. 2	*Leptostylus* sp. 2	3	0.9	2.5	2.4	30.01
Mec_anten	*Mecotetartus antennatus* (Bates, 1872)	2	0.1	0.1	2	59.82
Met_pyrrh	*Metaleptus pyrrhulus* (Bates, 1880)	1	1	1	1	30.86
Neo_cacic	*Neoclytus cacicus* (Chevrolat, 1860)	1	0.4	0.1	1	34.25
Neo_trili	*Neoptychodes trilineatus* (Linnaeus, 1771)	1	1	1	1	181.3
Ole_serri	*Olenosus serrimanus* (Bates, 1872)	1	0.4	0.1	1	75.56

Pla_diffi	*Placosternus difficilis* (Chevrolat, 1862)	1	0	0	1	54.4
Pol_hespe	*Poliaenus hesperus* (Chemsak & Linsley, 1988)	1	0.6	0.3	1	25.04
Pse_subar	*Pseudoperiboeum subarmatum* (Linsley, 1935)	1	1	1	1	57.27
Sph_picti	*Sphaenothecus picticornis* (Bates, 1880)	1	0.4	0.1	1	84.92
Sph_trili	*Sphaenothecus trilineatus* (Dupont, 1838)	1	0.6	0.3	1	111
Ste_anoma	*Steirastoma anomala* (Bates, 1880)	1	0.9	0.9	1	126.3
Ste_cribr	*Stenosphenus cribripennis cribripennis* (Thomson, 1860)	2	1	2	1.3	35.94
Tar_truqu	*Taricanus truquii* (Thomson, 1868)	1	0.4	0.1	1	104.9

Dry canopy

ID	Beetle species	Degree	d	SS	EP	BSI
Aca_lacry	*Acanthoderes lacrymans* (Thomson, 1865)	1	0.8	0.5	1	67.45
Aca_sp. 5	Acantocinini sp. 5	1	0.9	0.6	1	24.12
Aca_sp. 1	Acantocinini sp. 1	1	1	1	1	23.47
Aeg_albos	*Aegomorphus albosignus* (Chemsak & Noguera, 1993)	2	0.8	0.7	1.6	62.31
Ane_sp. 3	*Anelaphus* sp. 3	2	0.4	0.2	1.8	38.85
Ane_piceu	*Anelaphus piceum* (Chemsak, 1962)	3	0.7	2.1	2.8	48.39
Chy_dejea	*Chyptodes dejeani* (Thomson, 1865)	1	0.6	0.2	1	183.9
Col_sinal	*Colobothea sinaloensis* (Giesbert, 1979)	1	0.7	0.3	1	45.82
Ely_grand	*Elytroleptus grandis* (Linsley, 1935)	2	0.7	0.6	1.9	51.6
Eud_basim	*Euderces basimaculatus* (Giesbert & Chemsak, 1997)	7	0.6	2.9	6.6	11.54
Eud_pulch	*Euderces pulchra* (Bates, 1874)	3	0.9	2.3	2.1	31.61
Eud_sp. 1	*Euderces* sp. 1	1	0.9	0.7	1	19.98
Eup_sp	*Eupogonius* sp.	1	1	1	1	21.29
Eut_comus	*Eutrichillus comus* (Bates, 1881)	23	0.6	15	10	27.42
Hap_mandi	*Haplidus mandibularis* (Chemsak & Linsley, 1963)	1	0.2	0	1	31.86
Lag_arane	*Lagocheirus araneiformis ypsilon* (Voet, 1778)	1	0.1	0	1	155.8
Lag_obsol	*Lagocheirus obsoletus obsoletus* (Thomson, 1860)	8	0.5	2.6	4.7	105.1
Lep_sp. 3	*Leptostylus* sp. 3	2	0.4	0.3	1.9	29.58
Lep_sp. 2	*Leptostylus* sp. 2	3	0.4	0.3	3	30.01
Lep_sp. 1	*Leptostylus* sp. 1	1	0.2	0	1	33.05
Mec_anten	*Mecotetartus antennatus* (Bates, 1872)	4	0.5	1.2	2.4	59.82
Neo_cacic	*Neoclytus cacicus* (Chevrolat, 1860)	2	0.9	1.8	2	34.25
Neo_punct	*Neocompsa puncticollis asperula* (Bates, 1885)	2	0.8	0.8	1.4	29.65
Neo_sp. 1	*Neocompsa* sp. 1	1	0.2	0	1	34.17
Ole_serri	*Olenosus serrimanus* (Bates, 1872)	1	0.4	0.1	1	75.56
Ore_sp. 1	*Oreodera* sp. 1	1	0.3	0.1	1	41.05
Ore_brail	*Oreodera brailovskyi* (Chemsak & Noguera, 1993)	2	0.7	1.1	2	70.15
Pog_sp. 1	Pogonocherini sp. 1	1	0.2	0	1	69.75
Psa_borre	*Psapharochrus borrei* (Dugés, 1885)	4	0.8	2	2.1	59.94
Pse_subar	*Pseudoperiboeum subarmatum* (Linsley, 1935)	1	1	1	1	57.27
Rho_tenui	*Rhopalophora tenuis* (Chevrolat, 1855)	4	0.9	3.5	2.4	17.42
Sph_trili	*Sphaenothecus trilineatus* (Dupont, 1838)	4	0.9	2.2	3.6	84.92
Sph_picti	*Sphaenothecus picticornis* (Bates, 1880)	2	0.7	0.6	1.8	111
Ste_anoma	*Steirastoma anomala* (Bates, 1880)	1	0.8	0.6	1	126.3
Ste_sp	*Stenolis* sp.	1	0.7	0.3	1	20.7
Tar_ truq	*Taricanus truquii* (Thomson, 1868)	1	0.3	0.1	1	104.9
Tet_pictu	*Tetrasarus pictulus* (Bates, 1880)	2	0.2	0.1	2	36.87

Tri_sp	*Trichophoroides* sp.	1	0.4	0.1	1	62.47
Urg_sp. 4	*Urgleptes* sp. 4	1	0.7	0.3	1	7.741
Urg_sp. 2	*Urgleptes* sp. 2	1	0.3	0.1	1	4.886

Dry ground

ID	Beetle species	Degree	d	SS	EP	BSI
Aca_lacry	*Acanthoderes lacrymans* (Thomson, 1865)	3	0.9	1.6	2.6	67.45
Aca_sp. 1	Acantocinini sp. 1	1	0.8	0.5	1	24.12
Aca_sp. 3	Acantocinini sp. 3	1	0.9	0.8	1	25.11
Aca_sp. 5	Acantocinini sp. 5	1	0.5	0.1	1	23.47
Aca_sp. 6	Acantocinini sp. 6	1	0	0	1	15.75
Aeg_albos	*Aegomorphus albosignus* (Chemsak & Noguera, 1993)	5	0.6	1.8	3.7	62.31
Ane_piceu	*Anelaphus piceum* (Chemsak, 1962)	1	0.7	0.3	1	38.85
Atr_imple	*Atrypanius implexus* (Erichson, 1847)	1	0.2	0	1	29.76
Chy_dejea	*Chyptodes dejeani* (Thomson, 1865)	1	0.2	0.1	1	183.9
Ely_grand	*Elytroleptus grandis* (Linsley, 1935)	1	0.9	0.7	1	51.6
Eud_basim	*Euderces basimaculatus* (Giesbert & Chemsak, 1997)	1	1	1	1	11.54
Eud_pulch	*Euderces pulchra* (Bates, 1874)	3	1	3	2.6	31.61
Eup_sp	*Eupogonius* sp.	1	1	1	1	21.29
Eut_comus	*Eutrichillus comus* (Bates, 1881)	24	0.3	12	13	27.42
Lag_arane	*Lagocheirus araneiformis ypsilon* (Voet, 1778)	2	0.5	0.4	1.5	155.8
Lag_obsol	*Lagocheirus obsoletus obsoletus* (Thomson, 1860)	10	0.4	4.6	7.4	105.1
Lep_sp. 1	*Leptostylus* sp. 1	1	0.8	0.3	1	29.58
Lep_sp. 2	*Leptostylus* sp. 2	1	0.8	0.3	1	30.01
Loc_cornu	*Lochmaeocles cornuticeps cornuticeps* (Schaeffer, 1906)	1	0.4	0.1	1	97.96
Mec_anten	*Mecotetartus antennatus* (Bates, 1872)	2	0.5	0.7	1.8	59.82
Neo_trili	*Neoptychodes trilineatus* (Linnaeus, 1771)	1	0.9	0.8	1	181.3
Ole_serri	*Olenosus serrimanus* (Bates, 1872)	1	0.5	0.2	1	75.56
Ore_copei	*Oreodera copei* (McCarty, 2001)	2	0.8	0.9	1.9	45.19
Pha_marya	*Phaea maryannae* (Chemsak, 1977)	1	0.8	0.5	1	32.63
Pol_hespe	*Poliaenus hesperus* (Chemsak & Linsley, 1988)	1	0.1	0	1	25.04
Psa_borre	*Psapharochrus borrei* (Dugés, 1885)	2	0.9	1.1	1.6	59.94
Rho_tenui	*Rhopalophora tenuis* (Chevrolat, 1855)	1	0.8	0.5	1	17.42
Sph_picti	*Sphaenothecus picticornis* (Bates, 1880)	3	0.6	1	2.8	84.92
Sph_trili	*Sphaenothecus trilineatus* (Dupont, 1838)	3	0.8	1.7	2.7	111
Ste_anoma	*Steirastoma anomala* (Bates, 1880)	1	0.9	0.8	1	126.3
Ste_cribr	*Stenosphenus cribripennis cribripennis* (Thomson, 1860)	3	0.9	1.8	1.7	35.94
Tar_truqu	*Taricanus truquii* (Thomson, 1868)	1	1	1	1	104.9
Tet_pictu	*Tetrasarus pictulus* (Bates, 1880)	3	0.7	0.9	2.1	36.87
Tri_sp	*Trichophoroides* sp.	1	1	1	1	62.47
