# Supplementary material for: Spatio-temporal variation of Cerambycidae-host tree interaction networks
Source: PLoS One. 2020 Feb 10;15(2):e0228880. doi: 10.1371/journal.pone.0228880 (PMC7010308; doi:10.1371/journal.pone.0228880)
Supplement: S2 Table — Degree (number of interactions), d (species specialization), Species strength (SS), Effective partners (EP) and wood traits: Wood hardness (WH) and wood degradation (WD). (PDF) [file pone.0228880.s004.pdf]

Supporting information

S2 Table. Tree host values of tree-beetle network parameters per treatment in a tropical dry forest. Degree (number of interactions), d (species specialization), Species strength (SS), Effective partners (EP) and wood traits: Wood hardness (WH) and wood degradation (WD).

Rain canopy

Family	ID	Host tree	Degree	d	SS	EP	WH	WD
Anacardiaceae	Amp_adstr	*Amphipterygium adstringens* (Schltdl.) Standl.	2	0.01	0.15	1.57	0.61	0.08
Malpighiaceae	Bun_canes	*Bunchosia canescens* (Aiton) DC.	1	0.92	0.78	1.00	0.69	0.17
Burseraceae	Bur_copal	*Bursera copallifera* (DC.) Bullock	1	0.00	0.01	1.00	0.51	0.11
Burseraceae	Bur_grand	*Bursera grandifolia* (Schltdl.) Engl.	5	0.68	3.68	4.13	0.42	0.12
Malvaceae	Cei_aescu	*Ceiba aesculifolia* (Kunth) Britten & Baker f.	2	0.45	1.05	1.99	0.41	0.09
Fabaceae	Con_multi	*Conzattia multiflora* (B.L. Rob.) Standl.	2	0.50	0.67	1.89	0.50	0.14
Fabaceae	Dip_robin	*Diphysa robinioides* Benth. & Oerst.	1	0.77	0.33	1.00	0.86	0.23
Fabaceae	Dip_suber	*Diphysa suberosa* S.Watson	2	0.42	1.01	2.00	0.75	0.23
Moraceae	Fic_cotin	*Ficus cotinifolia*Kunth	2	0.00	0.14	1.57	0.54	0.13
Moraceae	Fic_croca	*Ficus crocata* (Miq.) Mart. ex Miq.	2	0.86	1.10	1.61	0.51	0.13
Moraceae	Fic_pertu	*Ficus pertusa* L.f.	3	0.28	1.37	1.86	0.69	0.21
Moraceae	Fic_petio	*Ficus petiolaris* Kunth	1	0.90	0.67	1.00	0.61	0.16
Convolvulaceae	Ipo_pauci	*Ipomoea pauciflora* M. Martens & Galeottia	4	0.75	2.84	2.93	0.35	0.10
Fabaceae	Lys_acapu	*Lysiloma acapulcense* (Kunth) Benth.	1	1.00	1.00	1.00	0.72	0.24
Fabaceae	Lys_divar	*Lysiloma divaricatum* (Jacq.) J.F.Macbr.	2	0.52	0.28	2.00	0.97	0.21
Fabaceae	Leu_escul	*Leucaena esculenta* (DC.) Benth.	3	0.24	1.36	2.59	0.73	0.23
Fabaceae	Leu_sp. 1	*Leucaena* sp. 1	2	0.26	1.02	1.60	0.51	0.14
Fabaceae	Mim_benth	*Mimosa benthamii* J.F.Macbr.	1	0.00	0.01	1.00	0.78	0.28
Malpighiaceae	Mal_mexic	*Malpighia mexicana* A. Juss.	1	0.63	0.17	1.00	0.77	0.15
Euphorbiaceae	Sap_macro	*Sapium macrocarpum* Müll.Arg.	2	0.29	0.59	1.19	0.44	0.10
Bignoniaceae	Tec_stans	*Tecoma stans* (L.) Juss. ex Kunth	2	0.88	1.11	1.57	0.74	0.25
Fabaceae	Van_farne	*Vachellia farnesiana* Wight & Arn.	2	0.93	1.67	1.89	0.83	0.15

Rain ground

Family	ID	Host tree	Degree	d	SS	EP	WH	WD
Anacardiaceae	Amp_adstr	*Amphipterygium adstringens* (Schltdl.) Standl.	2	0.56	1.13	1.75	0.61	0.08

Burseraceae	Bur_bipin	*Bursera bipinnata* (Moç. & Sessé ex DC.) Engl.	3	0.54	2.02	2.87	0.50	0.16
Malpighiaceae	Bun_canes	*Bunchosia canescens* (Aiton) DC.	1	1.00	1.00	1.00	0.69	0.17
Burseraceae	Bur_copal	*Bursera copallifera* (DC.) Bullock	4	0.55	2.45	3.71	0.51	0.11
Burseraceae	Bur_fagar	*Bursera fagaroides* (Kunth) Engl.	1	0.76	0.33	1.00	0.53	0.11
Burseraceae	Bur_grand	*Bursera grandifolia* (Schltdl.) Engl.	3	0.20	0.78	2.23	0.42	0.12
Burseraceae	Bur_lanci	*Bursera lancifolia* (Schltdl.) Engl.	1	0.00	0.01	1.00	0.51	0.18
Malvaceae	Cei_aescu	*Ceiba aesculifolia* (Kunth) Britten & Baker f.	2	0.79	1.03	1.46	0.41	0.09
Anacardiaceae	Com_macro	*Comocladia macrophylla* (Hook. & Arn.) L.Riley	1	0.36	0.09	1.00	0.41	0.10
Fabaceae	Con_multi	*Conzattia multiflora* (Robinson) Standl.	4	0.51	3.18	2.91	0.50	0.14
Fabaceae	Dip_robin	*Diphysa robinioides* Benth. & Oerst.	1	0.37	0.06	1.00	0.86	0.23
Fabaceae	Ery_ameri	*Erythrina americana* Mill.	1	0.00	0.01	1.00	0.32	0.12
Fabaceae	Eys_polys	*Eysenhardtia polystachya* (Ortega) Sarg.	1	1.00	1.00	1.00	0.75	0.26
Euphorbiaceae	Eup_schle	*Euphorbia schlechtendalii* Boiss.	5	0.34	1.68	3.92	0.56	0.16
Moraceae	Fic_cotin	*Ficus cotinifolia* Kunth	2	0.12	0.12	1.46	0.54	0.13
Moraceae	Fic_croca	*Ficus crocata* (Miq.) Mart. ex Miq.	1	1.00	1.00	1.00	0.51	0.13
Moraceae	Fic_pertu	*Ficus pertusa* L.f.	1	0.00	0.01	1.00	0.69	0.21
Moraceae	Fic_petio	*Ficus petiolaris* Kunth	1	0.00	0.01	1.00	0.61	0.16
Convolvulaceae	Ipo_pauci	*Ipomoea pauciflora* M. Martens & Galeotti	1	0.82	0.57	1.00	0.35	0.10
Fabaceae	Leu_escul	*Leucaena esculenta* (DC.) Benth.	1	0.05	0.03	1.00	0.73	0.23
Fabaceae	Leu_sp. 1	*Leucaena* sp. 1	4	0.48	2.88	1.85	0.51	0.14
Urticaceae	Myr_cordi	*Myriocarpa cordifolia* Liebm.	1	0.42	0.07	1.00	0.38	0.12
Malpighiaceae	Mal_mexic	*Malpighia mexicana* A. Juss.	1	0.00	0.01	1.00	0.77	0.15
Malpighiaceae	Mal_sp. 1	Malpighiaceae sp. 1	1	0.00	*0.01*	1.00	0.61	0.20
Euphorbiaceae	Sap_macro	*Sapium macrocarpum* Müll.Arg.	2	0.64	0.53	1.31	0.44	0.10
Fabaceae	Van_farne	*Vachellia farnesiana* Wight & Arn.	1	0.98	0.94	1.00	0.83	0.15
Lamiaceae	Vit_molli	*Vitex mollis* Kunth	1	1.00	1.00	1.00	0.49	0.21
Celastraceae	Wim_confu	*Wimmeria confusa* Hemsl.	1	0.05	0.03	1.00	0.77	0.26

Dry canopy

Family	ID	Host tree	Degree	d	SS	EP	WH	WD
Anacardiaceae	Amp_adstr	*Amphipterygium adstringens* (Schltdl.) Standl.	2	0.15	0.14	1.89	0.61	0.08
Annonaceae	Ann_cheri	*Annona cherimola* Mill.	4	0.75	2.66	1.96	0.54	0.11
Annonaceae	Ann_squam	*Annona squamosa* L.	3	0.27	1.04	2.60	0.48	0.18

Burseraceae	Bur_bipin	*Bursera bipinnata* (Moç. & Sessé ex DC.) Engl.	1	0.09	0.03	1.00	0.50	0.16
Malpighiaceae	Bun_canes	*Bunchosia canescens* (Aiton) DC.	1	0.46	0.08	1.00	0.69	0.17
Burseraceae	Bur_copal	*Bursera copallifera* (DC.) Bullock	6	0.33	3.37	3.80	0.51	0.11
Burseraceae	Bur_fagar	*Bursera fagaroides* (Kunth) Engl.	3	0.16	0.12	3.00	0.53	0.11
Burseraceae	Bur_grand	*Bursera grandifolia* (Schltdl.) Engl.	3	0.34	1.09	2.45	0.42	0.12
Malvaceae	Cei_aescu	*Ceiba aesculifolia* (Kunth) Britten & Baker f.	6	0.67	2.95	3.78	0.41	0.09
Cannabaceae	Cel_cauda	*Celtis caudata* Planch.	2	0.43	1.01	2.00	0.77	0.19
Asteraceae	Cri_hebeb	*Critonia hebebotrya* DC.	1	1.00	1.00	1.00	0.61	0.17
Fabaceae	Con_multi	*Conzattia multiflora* (Robinson) Standl.	1	0.09	0.03	1.00	0.50	0.14
Meliaceae	Ced_salva	*Cedrela salvadorensis* Standl.	1	0.41	0.04	1.00	0.50	0.11
Fabaceae	Dip_robin	*Diphysa robinioides* Benth. & Oerst.	1	0.41	0.04	1.00	0.86	0.23
Fabaceae	Dip_suber	*Diphysa suberosa* S.Watson	2	0.86	0.95	1.25	0.75	0.23
Fabaceae	Ery_ameri	*Erythrina americana* Mill.	4	0.44	2.24	2.89	0.32	0.12
Fabaceae	Ent_cyclo	*Enterolobium cyclocarpum* (Jacq.) Griseb.	1	0.58	0.11	1.00	0.62	0.12
Moraceae	Fic_cotin	*Ficus cotinifolia* Kunth	1	0.17	0.06	1.00	0.62	0.12
Moraceae	Fic_pertu	*Ficus pertusa* L.f.	2	0.30	1.01	1.89	0.69	0.21
Malvaceae	Gua_ulmif	*Guazuma ulmifolia* Lam.	3	0.78	0.98	1.65	0.59	0.17
Fabaceae	Hae_brasi	*Haematoxylum brasiletto* H.Karst.	3	0.60	0.59	3.00	1.04	0.24
Malvaceae	Hel_tereb	*Heliocarpus terebinthinaceus* (DC.) Hochr.	1	0.74	0.25	1.00	0.67	0.22
Convolvulaceae	Ipo_arbor	*Ipomoea arborescens* (Humb. & Bonpl. ex Willd.) G. Don	3	0.79	1.18	2.38	0.46	0.14
Convolvulaceae	Ipo_muroc	*Ipomoea murucoides* Roem. & Schult.	6	0.63	3.33	3.93	0.35	0.08
Convolvulaceae	Ipo_pauci	*Ipomoea pauciflora* M. Martens & Galeotti	1	0.49	0.07	1.00	0.35	0.10
Juglandaceae	Jug_sp. 1	*Juglans* sp. 1	1	0.00	0.01	1.00	0.27	0.14
Fabaceae	Lys_acapu	*Lysiloma acapulcense* (Kunth) Benth.	3	0.90	2.12	2.06	0.72	0.24
Fabaceae	Lys_divar	*Lysiloma divaricatum* (Jacq.) J.F.Macbr.	2	0.22	0.15	2.00	0.97	0.21
Malpighiaceae	Las_salic	*Lasiocarpus salicifolius* Liebm.	1	0.87	0.50	1.00	0.76	0.25
Fabaceae	Leu_sp. 1	*Leucaena* sp. 1	1	0.00	*0.01*	1.00	0.51	0.14
Araliaceae	Ore_pelta	*Oreopanax peltatus* Linden ex Regel	1	0.09	0.03	1.00	0.61	0.17
Oleaceae	Ole_sp	Oleaceae sp.	2	0.10	0.06	2.00	0.77	0.14
Celastraceae	Pri_celas	*Pristimera celastroides* (Kunth) A.C. Sm.	3	0.89	1.78	2.60	0.68	0.24
Rosaceae	Pru_corta	*Prunus cortapico* Kerber ex Koehneo	1	0.58	0.11	1.00	0.65	0.17
Fabaceae	Pit_dulce	*Pithecellobium dulce* (Roxb.) Benth.	1	1.00	1.00	1.00	0.74	0.23

Malvaceae	Pse_ellip	*Pseudobombax ellipticum* (Kunth) Dugand	3	0.87	1.47	2.38	0.30	0.07
Fabaceae	Pte_orbic	*Pterocarpus orbiculatus* DC.	1	1.00	1.00	1.00	0.83	0.20
Asteraceae	Pit_praec	*Pittocaulon praecox* (Cav.) H.Rob. & Brettell	1	0.40	0.07	1.00	0.52	0.04
Apocynaceae	Plu_rubra	*Plumeria rubra* L.	2	0.02	0.03	1.89	0.36	0.03
Rubiaceae	Ran_echin	*Randia echinocarpa* Moc. & Sessé ex DC.	1	0.81	0.43	1.00	0.84	0.20
Sapotaceae	Sid_capir	*Sideroxylon capiri* (A.DC.) Pittier	2	0.83	1.04	1.96	0.74	0.14
Euphorbiaceae	Sap_macro	*Sapium macrocarpum* Müll.Arg.	3	0.40	0.93	1.31	0.44	0.10
Anacardiaceae	Spo_purpu	*Spondias purpurea* L.	5	0.47	3.55	3.17	0.44	0.13
Bignoniaceae	Tec_stans	*Tecoma stans* (L.) Juss. ex Kunth	1	0.74	0.25	1.00	0.74	0.25
Fabaceae	Van_farne	*Vachellia farnesiana* Wight & Arn.	2	0.55	0.38	1.99	0.83	0.15
Lamiaceae	Vit_molli	*Vitex mollis* Kunth	2	0.73	0.74	1.18	0.49	0.21
Fabaceae	Van_penna	*Vachellia pennatula* (Schltdl. & Cham.) Seigler & Ebinger	1	0.00	0.01	1.00	0.82	0.15

Dry ground

Family	ID	Host tree	Degree	d	SS	EP	WH	WD
Anacardiaceae	Amp_adstr	*Amphipterygium adstringens* (Schltdl.) Standl.	2	0.08	0.04	1.89	0.61	0.08
Annonaceae	Ann_cheri	*Annona cherimola* Mill.	1	0.88	0.60	1.00	0.54	0.11
Annonaceae	Ann_squam	*Annona squamosa* L.	2	0.71	1.03	1.75	0.48	0.18
Malpighiaceae	Bun_canes	*Bunchosia canescens* (Aiton) DC.	1	0.80	0.44	1.00	0.69	0.17
Burseraceae	Bur_copal	*Bursera copallifera* (DC.) Bullock	8	0.43	4.49	3.97	0.51	0.11
Burseraceae	Bur_fagar	*Bursera fagaroides* (Kunth) Engl.	3	0.21	0.41	2.53	0.53	0.11
Burseraceae	Bur_grand	*Bursera grandifolia* (Schltdl.) Engl.	4	0.37	1.35	3.91	0.42	0.12
Burseraceae	Bur_lanci	*Bursera lancifolia* (Schltdl.) Engl.	1	0.00	0.01	1.00	0.51	0.18
Malvaceae	Cei_aescu	*Ceiba aesculifolia* (Kunth) Britten & Baker f.	4	0.82	2.16	1.78	0.41	0.09
Asteraceae	Cri_hebeb	*Critonia hebebotrya* DC.	3	0.85	2.01	1.98	0.61	0.17
Fabaceae	Con_multi	*Conzattia multiflora* (Robinson) Standl.	1	0.27	0.09	1.00	0.50	0.14
Meliaceae	Ced_salva	*Cedrela salvadorensis* Standl.	1	0.85	0.56	1.00	0.50	0.11
Malvaceae	Cei_sp.1	*Ceiba sp.1*	3	0.63	2.01	3.00	0.38	0.10
Fabaceae	Dip_robin	*Diphysa robinioides* Benth. & Oerst.	2	0.18	0.09	2.00	0.86	0.23
Fabaceae	Ery_ameri	*Erythrina americana* Mill.	2	0.21	0.14	1.75	0.32	0.12
Moraceae	Fic_cotin	*Ficus cotinifolia* Kunth	2	0.08	0.04	1.89	0.54	0.13
Moraceae	Fic_croca	*Ficus crocata* (Miq.) Mart. ex Miq.	1	0.15	0.03	1.00	0.51	0.13
Moraceae	Fic_pertu	*Ficus pertusa* L.f.	2	0.28	0.13	2.00	0.69	0.21

Fabaceae	Hae_brasi	*Haematoxylum brasiletto* H.Karst.	1	0.53	0.11	1.00	1.04	0.24
Convolvulaceae	Ipo_arbor	*Ipomoea arborescens* (Humb. & Bonpl. ex Willd.) G. Don	2	0.90	1.13	1.65	0.46	0.14
Convolvulaceae	Ipo_pauci	*Ipomoea pauciflora* M. Martens & Galeotti	2	0.61	0.68	1.89	0.35	0.10
Juglandaceae	Jug_sp. 1	*Juglans* sp. 1	1	1.00	1.00	1.00	0.27	0.14
Fabaceae	Lys_acapu	*Lysiloma divaricatum* (Jacq.) J.F.Macbr.	1	1.00	1.00	1.00	0.72	0.24
Fabaceae	Leu_macro	*Leucaena macrophylla* Benth.	2	0.46	0.34	1.89	0.63	0.19
Malpighiaceae	Las_salic	*Lasiocarpus salicifolius* Liebm.	2	0.47	1.01	2.00	0.76	0.25
Fabaceae	Mim_benth	*Mimosa benthamii* J.F.Macbr.	1	0.84	0.50	1.00	0.78	0.28
Malpighiaceae	Mal_sp. 1	Malpighiaceae sp. 1	1	0.10	0.02	1.00	0.61	0.20
Fabaceae	Mim_galeo	*Mimosa galeottii* Benth.	3	0.91	2.01	1.71	0.85	0.21
Araliaceae	Ore_pelta	*Oreopanax peltatus* Linden ex Regel	1	0.25	0.07	1.00	0.61	0.17
Oleaceae	Ole_sp	Oleaceae sp.	2	0.24	0.22	1.89	0.77	0.14
Celastraceae	Pri_celas	*Pristimera celastroides* (Kunth) A.C. Sm.	2	0.89	1.25	1.89	0.68	0.24
Fabaceae	Pte_orbic	*Pterocarpus orbiculatus* DC.	2	0.71	1.08	2.00	0.83	0.20
Asteraceae	Pit_praec	*Pittocaulon praecox* (Cav.) H.Rob. & Brettell	2	0.38	0.29	1.65	0.52	0.04
Fagaceae	Que_glauc	*Quercus glaucoides* M.Martens & Galeotti	1	1.00	1.00	1.00	0.71	0.24
Rubiaceae	Ran_echin	*Randia echinocarpa* Moc. & Sessé ex DC.	2	0.76	0.70	1.89	0.84	0.20
Rubiaceae	Ran_sp. 1	*Randia* sp. 1	1	0.80	0.44	1.00	0.76	0.19
Euphorbiaceae	Sap_macro	*Sapium macrocarpum* Müll.Arg.	3	0.43	0.88	2.85	0.44	0.10
Anacardiaceae	Spo_purpu	*Spondias purpurea* L.	3	0.40	1.33	2.65	0.44	0.13
Apocynaceae	Tab_glabr	*Tabernaemontana glabra* (Benth.) A.O. Simões & M.E. Endress	2	0.47	1.01	2.00	0.42	0.20
Lamiaceae	Vit_molli	*Vitex mollis* Kunth	4	0.47	1.29	4.00	0.49	0.21
Fabaceae	Van_penna	*Vachellia pennatula* (Schltdl. & Cham.) Seigler & Ebinger	1	1.00	1.00	1.00	0.82	0.15
Celastraceae	Wim_confu	*Wimmeria confusa* Hemsl.	1	0.15	0.03	1.00	0.77	0.26
