# Supplementary material for: Spatio-temporal variation of Cerambycidae-host tree interaction networks
Source: PLoS One. 2020 Feb 10;15(2):e0228880. doi: 10.1371/journal.pone.0228880 (PMC7010308; doi:10.1371/journal.pone.0228880)
Supplement: S3 Table — A principal component analysis was performed for the insect species of the four treatments: Rain canopy (Rc), Rain ground (Rg), Dry canopy (Dc) and Dry ground (Dg) of tropical dry forest. In bold correlation values r > 0.60. (PDF) [file pone.0228880.s005.pdf]

Supporting information

S3 Table. Beetle principal component analysis. Correlation values of four variables with the two main principal components. A principal component analysis was performed for the insect species of the four treatments: Rain canopy (Rc), Rain ground (Rg), Dry canopy (Dc) and Dry ground (Dg) of tropical dry forest. In bold correlation values > 0.60.

	Rc		Rg		Dc		Dg	
	PC1	PC2	PC1	PC2	PC1	PC2	PC1	PC2
Number of interactions	**0.99**	0.08	**0.99**	0.11	**0.97**	0.13	**0.97**	-0.08
Species level specialization	-0.19	**0.72**	-0.14	**0.75**	0.09	**-0.78**	-0.43	-0.40
Effective partners	**0.99**	0.04	**0.98**	0.14	**0.98**	0.11	**0.97**	-0.05
Body size index	-0.01	**-0.78**	-0.20	**0.71**	-0.24	**0.71**	-0.03	**0.92**
Eigenvalue	2.00	1.15	2.01	1.12	1.98	1.15	2.10	1.02
Variance explained (%)	50.06	28.80	50.42	28.00	49.54	28.83	52.53	25.71
Cumulative variance explained (%)	50.06	78.86	50.42	78.42	49.54	78.38	52.53	78.24
