# Supplementary material for: Spatio-temporal variation of Cerambycidae-host tree interaction networks
Source: PLoS One. 2020 Feb 10;15(2):e0228880. doi: 10.1371/journal.pone.0228880 (PMC7010308; doi:10.1371/journal.pone.0228880)
Supplement: S4 Table — A principal component analysis was performed for the tree host species of the four treatments: Rain canopy (Rc), Rain ground (Rg), Dry canopy (Dc) and Dry ground (Dg) of tropical dry forest. In bold correlation values r > 0.60. (PDF) [file pone.0228880.s006.pdf]

Supporting information

S4 Table. Host tree principal component analysis. Correlation values of four variables with the two main principal components. A principal component analysis was performed for the tree host species of the four treatments: Rain canopy (Rc), Rain ground (Rg), Dry canopy (Dc) and Dry ground (Dg) of tropical dry forest. In bold correlation values > 0.60.

	Rc		Rg		Dc		Dg	
	PC1	PC2	PC1	PC2	PC1	PC2	PC1	PC2
Number of interactions	**0.84**	0.46	**0.81**	0.48	**0.86**	0.43	**0.84**	0.44
Species strength	**0.79**	0.54	**0.77**	0.56	**0.79**	0.57	**0.75**	0.59
Wood hardness	**-0.81**	0.45	**-0.67**	**0.62**	**-0.73**	0.54	**-0.68**	0.59
Wood degradation	**-0.65**	**0.69**	**-0.71**	0.57	**-0.65**	**0.66**	**-0.69**	0.59
Eigenvalue	2.43	1.19	2.22	1.27	2.43	1.25	2.22	1.26
Variance explained (%)	60.80	29.95	55.51	31.92	58.70	31.28	55.54	31.66
Cumulative variance explained (%)	60.80	90.75	55.51	87.43	58.70	89.99	55.54	87.20
