# Supplementary material for: Spatio-temporal variation of Cerambycidae-host tree interaction networks
Source: PLoS One. 2020 Feb 10;15(2):e0228880. doi: 10.1371/journal.pone.0228880 (PMC7010308; doi:10.1371/journal.pone.0228880)
Supplement: Attachment — Submitted filename: Response to Reviewers PONE-D-19-19487.doc [file pone.0228880.s007.doc]

**Response to Reviews Comments to Manuscript PONE-D-19-19487**

| **Reviewer # 1 (PONE-D-19-19487)** | |  |  |
| --- | --- | --- | --- |
| **Page-line** | **Submitted manuscript Corrections / Comments** | **Page-line** | **Revised manuscript “Changes” / Clarification** |
| **General comments** | |  |  |
|  | My only, but serious, concern here is that the authors, perhaps blinded by numerical methods of analysis, misinterpret their data by concluding that their networks are “with a high specialization degree” as stated in Abstract and repeated in the text.  And yet, when you look at the actual food webs (Fig. 3) you can see that in all four food webs the most common cerambycid is always the same species, and that in all food webs it was reared from a large number of hosts, including always all three most used tree species. This pattern strongly suggests that the networks have actually very low degree of specialization, and that the high specialization values are caused by the predominance of rare species and interactions in the data, i.e. undersampling. |  | Thanks for your comments.  We carry out an experimental study where we offered the same resource (branches) in each of our treatments, therefore, we consider that our experimental design is suitable to describe the interaction patterns. In this sense, other authors who have worked with this type of interaction have described them as specialized at network level (index H2; ie, Wende et al., 2017; Ramírez-Hernández et al., 2019) it which agree with our results. In addition, this index of specialization has shown to be robust against the variation in sampling intensity, network size, and symmetry (Blüthgen et al., 2006). |
|  | Every time a species is common, it feeds on numerous hosts. The authors also found “a high turnover of cerambycid species and their interactions” among the four food webs. However, these are food webs referring to spatially nearby sites (ground and canopy), and temporally adjacent seasons (dry and wet). For relatively mobile and long-living beetles such as cerambycids, such turnover in species and interactions is again unlikely to reflect biological reality, but more likely undersampling. | L 469-473 | Our results show *E*. *commus* as a highly generalist species, the metric used in this study is at the level of the entire network, considering all interactions between the host tree and Cerambycidae, and not species-species. In this regard, Novotny and Basset (2005) mention that “*Although it is believed that larval host plant ranges are more restricted than those of adults… This is a notable problem in herbivorous beetles where larvae and adults often belong to different guilds.* Also specify that “*Concealed herbivore guilds are more specialized than those that feed externally on temperate vegetation*”*.*  Regarding the high turnover of species and interactions that we describe, we consider that it is not due to a subsampling, because other authors such as Lee et al., (2014) found a vertical stratification in this group and recently Martínez-Hernández et al., (2019) show that the species composition is different between seasons in a dry tropical forest. |
|  | Finally, the authors should better discuss the existing literature on cerambycid host specialization from the tropics. For instance, Tavakilian has provided some extensive data from (wet) Neotropics, and Novotny & basset (2005 Proc R Soc) review some more cerambycid data sets. | L414-419 | The suggestion was accepted. We added the reference Novotny & Basset, 2005 to discuss with our results. |
| **Reviewer # 2 (PONE-D-19-19487)** | |  |  |
| Lines 150-159 | I may not have understood, but I would like to know how the host plants were collected. At each period (dry and rainy) were the trees sampled and made available for the beetles, or was only one collection performed for all experiments? Regardless of the way, it was not clear to me how the variation in host tree availability occurred (Table 1 in the  results). Did all plant species chosen for the experiment have beetles emerged? |  | Sampling was carried out for one year to consider the temporary variation. The branches were placed in the treatments depending on their availability. Two cuts were made, one in the dry season and one in the rainy season (eight branches in each one). Four branches were left in the canopy and four on the ground, these were left exposed to the environment for 8 months, then every 2 months were pick up one branch from canopy and one of the ground in each season dry and rainy. Later they were collected and taken to the laboratory, where the insects emerged. However, not all branches emerged insects. |
| Lines 227-229 | What does nestedness mean biologically for the system studied? The same goes for modularity analysis. | L 228-234 | Thanks for your comments. Both nestedness and modularity mean that interactions of longhorn beetle has non-random pattern.  The nested pattern means that specialist interacts with some species that interact with a generalist. Modularity means that some longhorn interactions are in subgroups. |
|  | It is not clear to me why there are two sections to talk about network level metrics (Structure of Cerambycidae-host tree interaction networks and Network level) whereas specialization, for example, is a network-level index such as nestedness and modularity. | L 226 | The suggestion was accepted. We deleted the subheading “Structure of Cerambycidae-host tree interaction networks”. |
| Lines 231 and 234 | Why did the authors use two different null models to test the significance of the analyzed metrics? How do these null models work? For example, Patefield algorithm is a restrictive null model. This is a good model to control for Type I error but leaves us exposed to Type II error. | L 245-248 | Thanks for your comments. We did not use two different null models to test the significance of network metrics. In all cases, we used Patefield null model based in r2dtable algorithm. |
|  | Please explain in the text why the authors are selecting this null model for modularity metric and null.t.test for the other metrics. In addition, the null.t.test is considered a very rough null-model test. | L 245-249 | We know that there is not a perfect null model for test significance metrics, but in this case we used this model because fixes the network size as well as the marginal totals of each row and column, given by the species’ total frequencies of interaction.  To test the significance statistics for networks metrics we change “null.t.test” by “null model” function for WNODF, H_2_ and niche overlap metrics and for Q index we use Z-scores proposed by Dormman et al. (2009). |
| Lines 231-232 | Regarding the modularity analysis you used the QuanBiMo algorithm. Since many methods for calculating  modularity are now available, why did you choose this one? For example, Leger et al. (2015), Methods in Ecology and  Evolution, 6:474-481 compared different methods and found that the Stochastic block models was the best method to retrieve modules in weighted networks. Authors may consider include a justification of the selected algorithm in Methods Section. | L 234-237 | Thanks for your comments.  We change the method for calculating Modularity. |
|  | Even if the authors keep using an algorithm that maximizes modularity, because according to Leger et al. (2015) modularity maximization and one of the two variants of the Markov Chain Clustering algorithm (MCL1 / 10) were the models with the best performance after the stochastic block model, why didn't the authors calculate the recently implemented LPAwb + algorithm in the bipartite package? The LPAwb+ algotithm robustly identify partitions with high modularity scores, showing to be efficient for the detection of subgroups in ecological networks (see Beckett 2016: https://doi.org/10.1098/rsos.140536). | L 234-237 | Thanks for your comments. We review the paper of Beckett (2016) and we implemented DIRTLPA+ algorithm and reanalyze our data. However, our results were equal as when we used Quanbimo. |
| Line 235 | Which package was used to calculate network metrics? | L 261 | We clarify this in methods sections. |
| Line 394 | Note that nestedness means the existence of both generalist and specialist species in the networks. | L399-400 | We rephrase the sentence. |
| Figure 3 | The species names became too small it might be interesting to change the abbreviated names to numbers for both  beetles and plants. | Figure 3 | We improve the names of both beetle and plants in Figure 3. |
